# Гипотиреоз и старение: поиск протективных факторов

**DOI:** 10.14341/probl13156

**Published:** 2023-05-11

**Authors:** А. К. Ильющенко, Л. В. Мачехина, Е. Н. Дудинская

**Affiliations:** Российский геронтологический научно-клинический центр; Российский геронтологический научно-клинический центр; Российский геронтологический научно-клинический центр

**Keywords:** гипотиреоз, субклинический гипотиреоз, долголетие, старение, гормональная терапия, тиреотропный гормон

## Abstract

Поиск ключевых звеньев процесса старения — одна из основных задач в развитии гериатрии. Все больше исследований в последние годы посвящено изучению геропротективных механизмов, влиянию различных состояний и заболеваний на старение в целом. Особое значение имеют определение возрастных инволютивных процессов в организме человека, решение вопроса о том, являются ли они частью нормального старения или патологией, которую необходимо корректировать в пожилом возрасте. Важным возраст-ассоциированным процессом является изменение активности эндокринной системы, одним из примеров которого являются нарушения в работе щитовидной железы. Высокая распространенность гипотиреоза в пожилом возрасте объясняет актуальность данной темы и наш интерес к ее изучению. Нашей задачей было оценить влияние снижения функции щитовидной железы на течение заболеваний у пациентов пожилого возраста. Нами был проведен обзор литературы за последние 10 лет, посвященный терапии гипотиреоза у гериатрических пациентов, и выявлены наиболее актуальные проблемы, связанные с компенсацией данной патологии у пожилых пациентов. Были изучены данные о взаимосвязи гипотиреоза и основных гериатрических синдромов, особое внимание было уделено связи с когнитивными нарушениями и нарушениями эмоциональной сферы.

## Abstract (en)

Searching for aging key points is one of the main problems in geriatrics. More and more research in recent years has been devoted to the study of geroprotective mechanisms, the impact of various conditions and diseases on aging in general. Of particular importance is the determination of age-related involutive processes in the human body, whether they are part of normal aging or a condition that needs to be corrected to improve the functioning of organs and systems. An important mechanism of aging starts is a change in hormonal activity of endocrine glands, in particular in hormonal activity of thyroid. Frequency of hypothyroidism in advanced age explains relevance of the chosen topic. The aim of the review was to find out the role hypothyroidism in aging. The main task was to define, whether thyroid hormones decrease in older age was a protective factor or pathological process. A review of the literature over the past 10 years on subclinical treatment was carried out and we identified the most pressing issues associated with hypothyroidism and aging. We studied data on the relationship between hypothyroidism and major geriatric syndromes, with special attention paid to cognitive diseases and emotional disorders.

## ВВЕДЕНИЕ

Одним из основных направлений развития гериатрии является поиск протективных механизмов в старении с целью увеличения продолжительности и улучшения качества жизни, которые зависят не только от уровня и доступности медицины, но и от полноты понимания этих механизмов, от умения применять знания на практике.

Хотя старение — закономерный процесс для организма, цель многих научных исследований в последние годы — не только понять процесс старения, но и найти ключевые точки воздействия с целью геропротекции.

Гормоны щитовидной железы (ЩЖ) регулируют скорость метаболических процессов в организме, изменяют активность адренергической системы, влияют на периферическое сопротивление сосудов, усиливают гликогенолиз и гликогенез со специфической контринсулярной активностью [1][2]. В процессе старения меняется динамика многих процессов в ЩЖ: снижаются поглощение ЩЖ радиоактивного йода и секреция тироксина, замедляются метаболизм и клиренс тироксина, а также периферическая конверсия тироксина в трийодтиронин [1][2].

Наиболее распространенной патологией ЩЖ в пожилом возрасте является гипотиреоз, характеризующийся стойким дефицитом тиреоидных гормонов, а именно его субклиническая форма [1]. Понимание патогенеза данных изменений помогает компенсировать гипотиреоз у гериатрических пациентов с максимальной эффективностью и безопасностью.

## ТИРЕОТРОПНЫЙ ГОРМОН — ОДИН ИЗ ОСНОВНЫХ МАРКЕРОВ ГИПОТИРЕОЗА

Многие симптомы гипотиреоза в пожилом возрасте выражены неярко, некоторые из них (утомляемость, мышечная слабость, сухость кожи, склонность к запорам) расцениваются как признаки «нормального старения» [3].

Тиреотропный гормон (ТТГ) является основным маркером для диагностики гипотиреоза, референсные интервалы данного гормона имеют важное значение в определении тактики лечения. Обсуждая оптимальные уровни ТТГ в пожилом возрасте, следует обратиться к исследованиям по определению их референсных показателей. Исследование NHANES III, проведенное в США, и исследование SHIP здоровой популяции в Германии схожи в целях — определение среди здорового населения референсных показателей [4, 5].

Исследование NHANES III проходило с 1988 по 1994 гг. с целью разработки и формулировки национальных нормативов здоровья. В общую выборку не включались лица младше 12 лет с зобом, получавшие препараты, влияющие на ЩЖ, и беременные женщины. Кроме того, были исключены пациенты, принимающие препараты половых гормонов, литий, а также те, у кого определялись повышенные антитела к ЩЖ. В группе участников у 97,5% верхний предел ТТГ составил 3,5 мЕд/л [5][6].

Исследование SHIP включило контрольную группу из 1488 человек без ультразвуковых признаков увеличения ЩЖ, изменений эхогенности ЩЖ и узловых образований. Участникам исследования был проведен ряд лабораторных тестов для формирования референсных показателей здорового населения. При анализе полученных данных в исследовании для ТТГ верхний предел составил 2,2 мЕд/л [5][6].

Несмотря на результаты крупных когортных исследований, верхняя граница ТТГ не была скорректирована в соответствии с полученными значениями. После детальной оценки полученных результатов и их обсуждения в США National Institute for Health and Care Excellence (NICE) определил верхнюю границу ТТГ как 4,0 мЕд/л. Аргументы в пользу данного решения: а) отсутствие доказательств того, что назначение пациентам с уровнем ТТГ 2,5–4,0 мЕд/л (и даже 4,0–10,0 мЕд/л) терапии препаратами тироксина имеет какие-либо преимущества с позиции отдаленного прогноза, особенно в плане снижения смертности от сердечно-сосудистой патологии; б) отнесение 5% популяции, не имеющих каких-либо заболеваний, к больным гипотиреозом приведет к колоссальным финансовым затратам, а также к эмоционально-личностным расстройствам у данной категории [1][3].

В работах, направленных на изучение распространенности субклинического и манифестного гипотиреоза у пожилых, средний возраст исследуемой группы редко превышал 70 лет, а исследований с выборкой долгожителей крайне мало. На базе Российского геронтологического научно-клинического центра в рамках федеральной программы «Здоровое долголетие» проводилось комплексное медицинское гериатрическое обследование граждан старше 90 лет. Целью исследования было оценить функциональный, когнитивный и эмоциональный статусы долгожителей для определения объема необходимой помощи со стороны социальных служб и медицинского персонала, кроме того, был проведен анализ функции ЩЖ по данным лабораторного обследования [7].

По данным регистра супердолгожителей Москвы было набрано 82 человека в возрасте от 95 до 105 лет, медиана ТТГ составила 2,26 (1,8–3,6) мЕД/л (проанализированы данные 64 долгожителей). У большей части участников исследования ТТГ был в пределах референсных значений (50 человек, 78,1%), повышение уровня ТТГ было выявлено у 12 долгожителей (18,8%), у 2 (3,1%) отмечалось снижение уровня ТТГ до 0,07 мЕд/л. Полученные результаты могут указывать на необходимость дальнейшего исследования более крупных когорт долгожителей с целью определения индивидуального подхода терапии данной возрастной группы.

## ВЛИЯНИЕ ГИПОТИРЕОЗА НА ЛИПИДНЫЙ ОБМЕН

В пожилом возрасте липидный обмен играет важную роль в достижении компенсации многих заболеваний, в особенности сердечно-сосудистых. При субклиническом гипотиреозе отмечается повышение уровня липопротеина(а), содержащего молекулу аполипопротеина-А, соединенную с аполипопротеином-В-100 — компонентом липопротеинов очень низкой плотности, что придает липопротеинам(а) атерогенные и тромбогенные свойства [8].

В исследовании В. Asvold и соавт. 30 656 человек без заболеваний ЩЖ было изучено влияние гипотиреоза на липидный обмен. При анализе результатов было установлено, что нарушение липидного обмена при субклиническом гипотиреозе выражается в повышении уровней общего холестерина и холестерина липопротеинов низкой плотности (ЛПНП), а также в увеличении соотношения ЛПНП/липопротеинов высокой плотности (ЛПВП) и триглицеридов (ТГ)/ЛПВП. Было также установлено, что раннее начало заместительной терапии и достижение эутиреоидного состояния значительно улучшают соотношение ЛПНП/ЛПВП, что позволяет снизить риск сердечно-сосудистых заболеваний [9].

В исследовании М. Pandrc и соавт. был проведен анализ эффективности заместительной терапии при субклиническом гипотиреозе (определялся как уровень ТТГ 4–10 мЕд/л при нормальном значении свободного тироксина (Т4св) у 35 пациентов, средний возраст которых составил 51,6±15,4 года). При оценке результатов биохимического исследования после курса заместительной терапии было отмечено снижение таких показателей, как уровень гомоцистеина (р=0,03), гликированного гемоглобина (р=0,001), общего холестерина (р=0,001), триглицеридов (р=0,007), липопротеинов(а) (p<0,001) [10].

Таким образом, у пациентов с субклиническим гипотиреозом необходимо выявлять нецелевые значения показателей липидного обмена и корректировать их в соответствии с индивидуальными сердечно-сосудистыми рисками.

## ВЛИЯНИЕ ГИПОТИРЕОЗА НА УГЛЕВОДНЫЙ ОБМЕН

Нарушения углеводного обмена — одна из наиболее часто встречаемых патологий пожилого возраста. Мы провели анализ исследований по оценке влияния снижения функции ЩЖ на состояние пациентов с метаболическими нарушениями.

В проспективном популяционном когортном исследовании L. Chaker и соавт. были проанализированы показатели ТТГ, свТ4 среди участников в возрасте старше 65 лет. При анализе данных было получено, что более высокие уровни ТТГ и низкие уровни свТ4 ассоциированы с более высоким риском развития сахарного диабета 2 типа и прогрессией предиабета в диабет. Как манифестный, так и субклинический гипотиреоз оказались ассоциированы со снижением чувствительности тканей к инсулину и со сниженной толерантностью к глюкозе. Это может иметь значение в достижении компенсации различных нарушений углеводного обмена [11].

Таким образом, у пациентов с различными нарушениями углеводного обмена важно оценивать функцию ЩЖ. Достижение эутиреоидного состояния у данной группы может улучшать прогноз заболеваний.

## ГИПОТИРЕОЗ И СЕРДЕЧНО-СОСУДИСТЫЕ ЗАБОЛЕВАНИЯ

Одним из заболеваний, требующих особой осторожности в коррекции у пациентов с гипотиреозом, является ишемическая болезнь сердца (ИБС). Существует мнение, что гипотиреоз вследствие атерогенного действия способствует развитию и прогрессированию ИБС [2].

В исследовании N. Rodondi и соавт. была проведена оценка риска развития ИБС у пациентов с субклиническим гипотиреозом. Для оценки были использованы данные 25 977 пациентов на основании баз данных MEDLINE и EMBASE (с 1950 г. по 31 мая 2010 г.). При оценке результатов были получены данные, продемонстрировавшие, что субклинический гипотиреоз был ассоциирован с повышенным риском развития ИБС, особенно у пациентов с концентрацией ТТГ 10 мЕд/л и выше (табл. 1) [12].

У пациентов с ИБС необходимо оценивать гормональный статус и индивидуально определять подходы к компенсации заболеваний ЩЖ.

**Table:** Таблица 1. Риски развития ИБС в зависимости от уровня ТТГ в исследовании N. Rodondi [12]

Уровень ТТГ	Риск развития ИБС
4,5–6,9 мЕД/л	1,0
7–9,9 мЕД/л	1,17
Более 10 мЕд/л	1,89

## ГИПОТИРЕОЗ И ОСТЕОПОРОЗ

В пожилом возрасте заболевания опорно-двигательного аппарата встречаются значительно чаще, чем в молодом. Снижение уровня гормонов ЩЖ может нарушать обмен костной ткани за счет снижения активности остеобластов. На данный момент нет единого мнения о необходимости компенсировать гипотиреоз с целью профилактики заболеваний костной ткани [13]. Остеопороз является наиболее распространенным метаболическим заболеванием костей и характеризуется снижением минеральной плотности костной ткани, что приводит к повышенному риску переломов. В костной ткани рецепторы трийодтиронина (Т3) взаимодействуют с альфа-изоформой ядерного рецептора тиреоидного гормона в остеобластах, T3 стимулирует активность остеобластов (дифференциацию и пролиферацию, а также синтез и минерализацию костного матрикса). Тем не менее на данный момент в исследованиях не удалось выявить достоверной связи между возникновением гипотиреоза и частотой встречаемости остеопороза, связь между снижением уровня гормонов ЩЖ и повышенным риском переломов также не была установлена [14]. Необходимы дальнейшие исследования для определения необходимости коррекции гипотиреоза у пациентов с остеопорозом.

## ГИПОТИРЕОЗ И ГЕРИАТРИЧЕСКИЕ СИНДРОМЫ

Функциональное состояние ЩЖ может влиять на распространенность гериатрических синдромов, то есть возраст-ассоциированных клинических состояний, ухудшающих качество жизни и повышающих риск неблагоприятных исходов. У пациентов со сниженной функцией ЩЖ в пожилом возрасте чаще выявляются снижение когнитивных функций, тревожно-депрессивные симптомы, более высокая частота мальнутриции и саркопении [15].

Когнитивные нарушения в пожилом возрасте встречаются намного чаще, чем в молодом [2, 15]. Одной из причин снижения когнитивных функций у гериатрических пациентов может быть нарушение работы ЩЖ. Так, в исследовании S. Recker и соавт. оценивались уровни тревоги, депрессии, показатели самооценки качества жизни после заместительной терапии левотироксином при субклиническом гипотиреозе в группе молодых и пожилых пациентов. Исследователями проведена оценка динамики показателей качества жизни по опросникам SF-36 и ThyPRO, когнитивных функций после 6 мес терапии. При анализе данных было обнаружено, что в группе пациентов пожилого возраста было значимое улучшение когнитивных функций, улучшение качества жизни, снизились показатели тревоги и депрессии [16].

В исследовании G. Pasqualetti и соавт. был проведен метаанализ 13 исследований с целью оценки риска когнитивных нарушений и деменции в группе с субклиническим гипотиреозом относительно популяции в целом. Для пациентов моложе 75 лет была получена достоверная ассоциация субклинического гипотиреоза с повышением риска когнитивных нарушений (р=0,02), деменции (р<0,01). Полученные результаты могут иметь большое значение для восстановления когнитивного потенциала пациентов в пожилом возрасте [17].

В исследовании Mexican Health and Aging Study была выделена группа пожилых людей с субклиническим гипотиреозом (определялся как уровень ТТГ в диапазоне 4,5–10 мЕд/мл при нормальном уровне свТ4) и проведен анализ распространенности гериатрических синдромов внутри данной группы. Наиболее распространенными гериатрическими синдромами у пациентов с субклиническим гипотиреозом оказались падения, депрессия и синдром старческой астении (табл. 2). Частота распространенности гериатрических синдромов оказалась выше в сравнении с группой пожилых пациентов с эутиреозом [18].

**Table:** Таблица 2. Результаты исследования Mexican Health and Aging Study по оценке наиболее распространенных гериатрических синдромов у пожилых пациентов с субклиническим гипотиреозом [19]

Гериатрический синдром	Отношение шансов	Доверительный интервал	p-критерий
Падения	1,79	1,16–2,77	0,0116
Старческая астения	2,17	1,40–3,38	0,0348
Депрессия	1,70	1,06–2,71	0,0246

Так, специалистам, работающим с пациентами с синдромом старческой астении, важно оценивать состояние ЩЖ для уменьшения выраженности различных гериатрических синдромов.

## ТЕРАПИЯ ГИПОТИРЕОЗА У ПОЖИЛЫХ ПАЦИЕНТОВ

Назначение гормональной заместительной терапии в пожилом возрасте у пациентов с гипотиреозом имеет свои особенности в сравнении с общей популяцией. Титрация дозы левотироксина требует большего времени и меньшего шага повышения дозы препаратов. Рассчитывая дозу левотироксина у больных с тяжелыми сопутствующими патологиями, следует начинать с минимальных доз (6,25–12,5 мкг), увеличивая дозу на 25 мкг каждые 2 мес до нормализации уровня ТТГ [1][3][4]. Такая осторожная титрация дозы позволяет пожилым пациентам лучше переносить терапию. Существует мнение, что при трудности компенсации гипотиреоза у пожилых пациентов показатель ТТГ может быть в пределах, не превышающих значений, характерных для субклинического гипотиреоза (не выше 10 мЕД/л), а период подбора оптимальной дозы — достигать 6 мес [19][20].

Таким образом, для пациентов пожилого возраста оптимальной дозой левотироксина является не та, которая полностью восстанавливает нормальный уровень Т4 и ТТГ в сыворотке, а та, которая смягчает симптоматику гипотиреоза и не влияет на течение сопутствующих заболеваний, не вызывает побочных эффектов [20][21].

Полисистемное влияние гипотиреоидного статуса на пожилого пациента, возможное ухудшение течения сопутствующих заболеваний требует своевременного выявления снижения функции ЩЖ и тщательного подбора оптимальной терапии данного состояния.

## ЗАКЛЮЧЕНИЕ

До сих пор нет единого мнения о том, какую роль снижение функции ЩЖ играет в процессе старения, однако нельзя считать субклинический гипотиреоз закономерным процессом в пожилом возрасте. Коррекция данной патологии у пожилых пациентов требует индивидуального подхода, цели терапии должны выбираться специалистом в зависимости от сопутствующих заболеваний, наличия сердечно-сосудистых, метаболических заболеваний, степени их компенсации. Необходимо разработать алгоритмы компенсации гипотиреоза в пожилом возрасте, в том числе в разных возрастных подгруппах пациентов старше 60 лет. Для решения данной задачи требуется проведение дальнейших исследований эффективности и безопасности терапии субклинического гипотиреоза.

## ДОПОЛНИТЕЛЬНАЯ ИНФОРМАЦИЯ

Источники финансирования. Работа выполнена по инициативе авторов без привлечения финансирования.

Конфликт интересов. Авторы декларируют отсутствие явных и потенциальных конфликтов интересов, связанных с содержанием настоящей статьи.

Участие авторов. Ильющенко А.К. — существенный вклад в концепцию и дизайн исследования, получение, анализ данных или интерпретацию результатов, написание статьи; Мачехина Л.В. — существенный вклад в концепцию и дизайн исследования, получение, анализ данных или интерпретацию результатов, внесение в рукопись существенной (важной) правки с целью повышения научной ценности статьи; Дудинская Е.Н. — существенный вклад в концепцию и дизайн исследования, получение, анализ данных или интерпретацию результатов, внесение в рукопись существенной (важной) правки с целью повышения научной ценности статьи.

Все авторы одобрили финальную версию статьи перед публикацией, выразили согласие нести ответственность за все аспекты работы, подразумевающую надлежащее изучение и решение вопросов, связанных с точностью или добросовестностью любой части работы.
